# Full Genome Virus Detection in Fecal Samples Using Sensitive Nucleic Acid Preparation, Deep Sequencing, and a Novel Iterative Sequence Classification Algorithm

**DOI:** 10.1371/journal.pone.0093269

**Published:** 2014-04-02

**Authors:** Matthew Cotten, Bas Oude Munnink, Marta Canuti, Martin Deijs, Simon J. Watson, Paul Kellam, Lia van der Hoek

**Affiliations:** 1 Wellcome Trust Sanger Institute, Hinxton, United Kingdom; 2 Laboratory of Experimental Virology, Department of Medical Microbiology, Center for Infection and Immunity Amsterdam (CINIMA), Academic Medical Center of the University of Amsterdam, Amsterdam, The Netherlands; 3 Department of Infection, University College London, London, United Kingdom; University of California, San Francisco, United States of America

## Abstract

We have developed a full genome virus detection process that combines sensitive nucleic acid preparation optimised for virus identification in fecal material with Illumina MiSeq sequencing and a novel post-sequencing virus identification algorithm. Enriched viral nucleic acid was converted to double-stranded DNA and subjected to Illumina MiSeq sequencing. The resulting short reads were processed with a novel iterative Python algorithm SLIM for the identification of sequences with homology to known viruses. *De novo* assembly was then used to generate full viral genomes. The sensitivity of this process was demonstrated with a set of fecal samples from HIV-1 infected patients. A quantitative assessment of the mammalian, plant, and bacterial virus content of this compartment was generated and the deep sequencing data were sufficient to assembly 12 complete viral genomes from 6 virus families. The method detected high levels of enteropathic viruses that are normally controlled in healthy adults, but may be involved in the pathogenesis of HIV-1 infection and will provide a powerful tool for virus detection and for analyzing changes in the fecal virome associated with HIV-1 progression and pathogenesis.

## Introduction

There are 219 virus species known to infect humans; calculations based on the rate of virus discovery indicate that there may be 265 human virus species yet to be discovered [1]. The advances in deep sequencing processes provide an important new tool for the identification of novel viruses. Correct phylogenetic analysis and virus transmission studies are best performed with as much sequence information as possible. Given the relatively small genome sizes of most viral genomes and the increased sequencing depth now available with deep sequencing platforms, generating full genomes of novel viruses should become the standard for virus identification. The characterization of novel full viral genomes present in clinical, animal or environmental samples is important for diagnostics, for identifying unexpected pathogens and for discovering disease etiology. Sample origin can also influence complexity: fecal samples have significant bacterial and dietary content and the derived nucleic acids contain large amounts of bacterial, bacteriophage, and plant viral nucleic acids in the resulting sequence data complicating detection of mammalian viruses.

Fecal material is a useful place to seek mammalian viruses for a number of reasons. The high titers and stable virions of fecal viruses results in increased sequence recovery. Fecal viruses are often found in high titers further improving detection. In addition to the enteric viruses that actually replicate in the gut, the fecal compartment can contain respiratory viruses (e.g. coronavirus) and hepatitis viruses improving the range of detection. The utility of searching for novel viruses in this compartment is well documented and deep sequencing of fecal derived nucleic acids is a rich source of new viruses from bats [2], [3], [4], wild rodents [5], pigs on domestic farms [6], California sea lions[7], wild pigeons [8] and human fecal material [9] [10], [11]. Changes in the fecal virome may be an extremely important feature of AIDS pathogenesis and detailed characterization of the fecal virome may provide new understanding of HIV-1 pathogenesis [12]. Studies seeking virus in sewage [13], [14], may present different set of discovery challenges including concentration of diluted starting material, the mixture of material from multiple individuals and species found in sewage and the risk that virus genome assembly creates an artificial chimera. Algorithms to process deep sequencing data have been devised and these are providing important advances for virus detection [15], [16]. An assumption has been made that a single dominant host nucleic acid exists in the sample. This may be the case with some sample types, however fecal material may include bacterial, fungal, plant nucleic acid from either commensal organisms or diet.

These studies demonstrate that there are many useful ways to discover viruses in the fecal material using deep sequencing. All of the studies so far have used manual or batch BLAST searching to identify the resulting sequences. Although this is quite effective, it is time consuming work, both implementing the BLAST searches themselves or parsing the BLAST output to extract useful information. Furthermore, as sequencing technologies have improved (e.g. moving from 454 to Illumina platforms) the total number of sequence reads to be processed has increased 10 to 100 fold, further increasing the processing work. Furthermore, a common challenge facing all virus detection in fecal material is the high content of bacteria and dietary nucleic acid that can interfere with the detection of mammalian viruses. This bystander nucleic acid both consumes precious sequencing resources and can dominate the resulting sequence data. Methods that improve or simplify virus detection amidst large amounts of peripheral nucleic acid are needed. The work described here provides a computational solution to simplify virus detection amidst the increasing amount of sequence data now available.

The VIDISCA method was developed as a sensitive process for recovering viral nucleic acid from many types of samples and for efficient amplification and identification of viral sequences based on restriction digestion, ligation of common adapters and amplification with adapter specific primers. The method has been used successfully in a number of virus detection applications [17], [18], combined with 454 deep sequencing to identify pathogens [19] [20] [21] [22] and with autologous antibody capture to identify the immunogenic viruses within a sample [23]. The method presented here is a combination of sample processing using VIDISCA enrichment for particle-protected nucleic acid and conversion of RNA to double-stranded DNA (dsDNA) [19], [20] followed by Illumina MiSeq deep sequencing and SLIM iterative BLAST processing. The analysis of the larger quantities of sequence data generated by this method required a novel iterative classification process, SLIM which removes abundant sequences and facilitates identification of rarer viral sequences amidst large amounts of host or bystander sequences. The novel method, ViSeq, was used to document the viral content of human fecal samples from HIV-1 infected patients and to provide full genome catalog of viruses found in this compartment in late stage AIDS patients.

## Materials and Methods

### Sample origin

Fecal samples were obtained from a sample bank of HIV-1 infected adult patients with diarrhea, aged above 18 who visited the out-patients clinic at the Academic Medical Center in the years 1994–1995 [24]. Fecal samples were diluted 1∶3 in broth (containing nutrient broth no 2 supplied by Oxoid, 500 IU penicillin per ml, 500 μg streptomycin and 3 μg amphotericin B per ml. Cell debris, bacteria and mitochondria were removed from 110 μL of this fecal suspension with a 10 minute centrifugation at 10,000×g. Residual DNA was degraded with 20 U TURBO DNase (Ambion). Undigested nucleic acid (virion-protected) was extracted using the Boom method [25], with elution of nucleic acids performed in sterile water. A reverse transcription reaction with Superscript II (Invitrogen) was performed using non-ribosomal random hexamers [26]. Subsequently, second strand DNA synthesis was performed with 5 U of Klenow fragment. Nucleic acids were purified by phenol/chloroform extraction and ethanol precipitation.

### Sequencing

Illumina MiSeg library prep was performed by standard Illumina methods. Briefly, each sample was sheared and size fractionated to 400–500 bp in length, ligated to Illumina adapters with a unique barcode per sample, then PCR amplified and multiplexed with 10 samples per run and sequencing was performed with an Illumina MiSeq instrument to generate ca. 1–3 million 150 nt paired-end reads per sample. Residual adapter containing reads were removed and reads were trimmed from the 3′ to a median phred score of 30 and minimum length of 50 nt using QUASR [27]. The short read data have been deposited in the European Nucleotide Archive with the accession numbers ERR233412-ERR233431.

### Taxonomic analysis with Megan4

For each sample a random set of 100,000 reads was subjected to nucleotide BLAST analysis using a local instance of BLAST+ against the nr nucleotide database. Only hits with an expectation values < 0.001 were collected. A comparative taxonomic classification of the BLAST output was performed with MEGAN version 4.70.4 [28] and the MEGAN output was visualized as a heat map using Python scripts.

### Identification of virus reads: the SLIM algorithm

For each sample a random set of 100,000 reads was selected and processed through an iterative blast algorithm with the following features: the first read was subjected to a BLASTN search limiting search entries to 2000–500,000 bp in length. The first BLASTN match with an e values below 0.001 was identified, the GenBank entry for that BLAST hit was retrieved and all reads in the readset that map to this sequence were identified. The identity of the BLAST hit was used to classify these mapped reads as viral or non-viral. If the read returned no significant BLASTN hit, it was placed in the mystery bin. At the end of each round of BLAST/GenBank retrieval/mapping, all mapped or mystery reads were removed from the readset to generate a remaining-reads set which was passed into the next round. In this manner, abundant reads are classified and removed in the initial cycle of the processing. The SLIM algorithm is written in Python and is available upon request.

### Assembly of full virus genomes

Full virus genomes were prepared from the raw dataset in the following manner. Initially SLIM was used to identify the closest full virus genomes for each sample. All reads in each sample were then mapped to the closest full genome reference using BWA [29] to give an indication of the level of coverage in the data set. *De novo* genome assembly was performed using SPAdes [30]. The resulting contigs were mapped directly to the expected viral genomes using MUMmer [31]. In addition, the SPAdes-generated contigs were processed with SLIM to identify all possible viral contigs. Coverage for each full genome was determined by mapping all reads to the final *de novo* assembled genome and reporting the number of reads with Phred quality score of 30 or above for each position. Mean coverage values for all full genomes is shown in Table 1. The GenBank accession numbers for all new complete or nearly complete viral genomes are listed in Table 1.

**Table 1:** Sample details.

Sample	Total reads^1^	Known agent^2^	Complete viral genome assembled^3^	GenBank Accession number	Mean genome coverage^4^
1	1,212,092	Aichi virus			
2	156,518	Norovirus			
3	1,011,850	Norovirus	Adenovirus_Amsterdam_1995	KJ194509	7.41
4	952,694	Cosavirus	Cosavirus_Amsterdam_1994	KJ194505	64.85
5	1,257,100	Norovirus			
6	1,719,448	Norovirus			
7	1,012,842	Norovirus	NV_Amsterdam_1994	KJ194504	264.67
8	1,077,900	Norovirus			
9	1,279,402	Norovirus	HBV_Amsterdam_1_1994	KJ194506	1.5
10	12,044,856	IIAS virus^5^	HBV_Amsterdam_2_1994	KJ194508	4.79
11	3,347,066	Aichi virus	TTV_Amsterdam_1994	KJ194503	2.16
12	2,426,096	Norovirus			
13	2,533,908	Norovirus			
14	1,612,944	Norovirus	NV_Amsterdam_1_1995	KJ194500	24.88
15	2,474,006	NANB-1 virus			
16	1,614,452	Norovirus	NV_Amsterdam_2_1995	KJ194510	1263.51
17	3,344,548	Norovirus	Adenovirus_Amsterdam_1995	KJ194501	97.56
			HPV_Amsterdam_1995	KJ194499	2.14
			NV_Amsterdam_3_1995	KJ194507	3.15
			TTV_Amsterdam_1995	KJ194502	7.09
18	1,450,342	Norovirus			
19	1,380,360	Norovirus			
20	5,338,614	NANB-1 virus			

1 Total number of MiSeq reads after removal low quality, short or adapter containing reads.

2 Viruses known to be present in sample from preliminary analysis.

3 Complete or nearly complete genomes obtained with *de novo* assembly.

4 All reads mapped to *de novo* assembled genome using BWA [29] and the value reports the mean number of times each position was sequenced.

5 Immunodeficiency-Associated Stool virus (IASvirus) [43].

## Results

**Figure 1:**
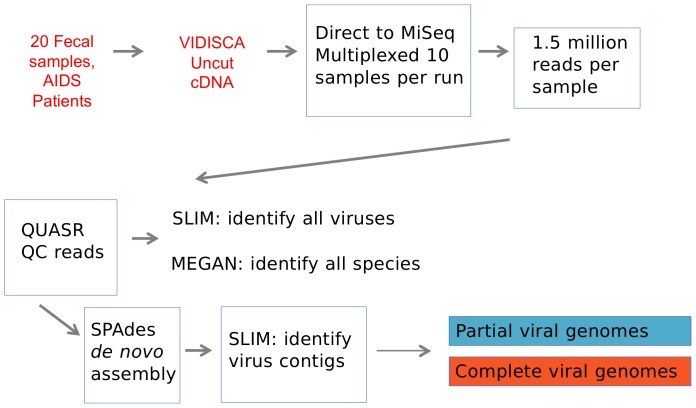
An overview of the ViSeq process.

**Figure 2:**
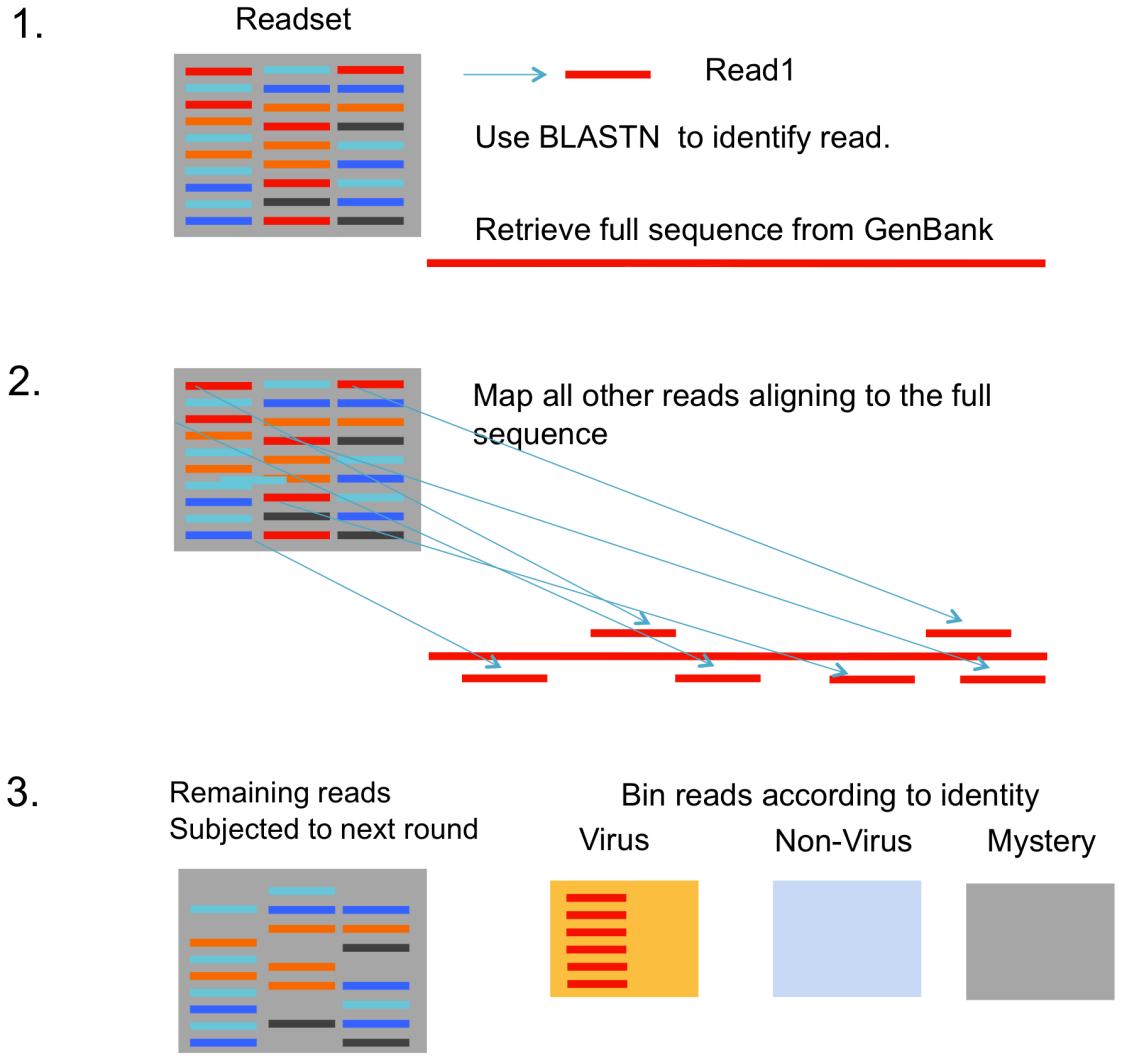
An overview of the SLIM read classification process.

We used the ViSeq method to analyze 20 fecal samples from HIV-1 infected individuals. An overview of the analysis process is presented in Figure 1. A modified VIDISCA method was used to harvest protected nucleic acid from the fecal material and to convert any RNA to cDNA using random-primed reverse transcription [19] [20]. The cDNA was converted into dsDNA and after phenol/chloroform extraction and ethanol precipitation, the cDNA was processed by shearing and adapter ligation into an Illumina library and subjected to MiSeq deep sequencing. The resulting sequences were processed to remove adapter sequences and trimmed from the 3′ end to a median Phred value of 30, resulting in a median value of 1.5×10^6^ reads/sample. Raw sequencing data was trimmed to remove low quality reads, analyzed for content using SLIM (Figure 2, see below) and subjected to de novo assembly followed by SLIM for additional content identification. Sample details and the yield of sequence data are provided in Table 1.

### Sensitivity of the ViSeq method

**Figure 3:**
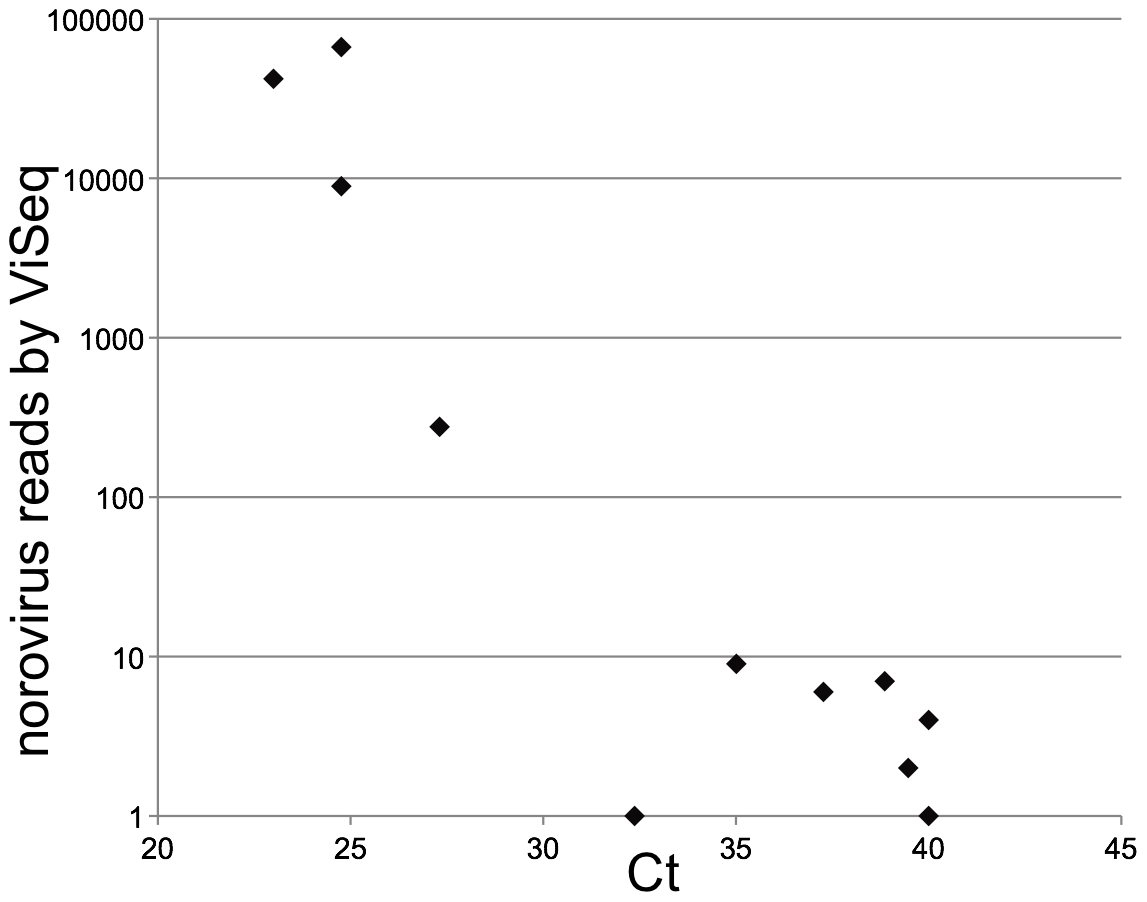
Detection of norovirus by real-time PCR vs the ViSeq process. Total ViSeq identified norovirus reads were compared to the Real time PCR determined norovirus viral loads. Pearson's correlation coefficient for all samples (−0.69), for all samples with Ct values below 35 (0.63) and for all samples with ViSeq reads above 10, (−0.59), indicate a strong negative correlation between the two methods of measurements.

To determine the sensitivity of ViSeq, the ability of the process to detect a pathogen known to be present in the fecal samples was assessed. Some samples contained norovirus at different genome loads as measured by real time PCR. The total number of norovirus reads in each sample was determined by ViSeq followed by mapping all reads to a close norovirus genome. This value was compared to the norovirus viral load determined by real-time PCR. Norovirus reads ranging from 1 to 42,000 were detected across the sample set and there was a strong correlation with the viral load measured by real time PCR (Figure 3). The cutoff for a positive clinical norovirus diagnosis with this real-time PCR assay is a threshold cycle (Ct) of 40; the ViSeq method detected norovirus with similar sensitivity, however without prior sequence knowledge required to design specific real-time PCR primers. Although deep sequencing is currently not a faster alternative to a virus specific real time PCR assay, it is clear that the two detection methods show similar levels of sensitivity for a known virus. Furthermore, the four samples with norovirus Ct values below 27 provided sufficient sequence coverage for full genome assembly (see below).

### General taxonomic classification of reads

**Figure 4:**
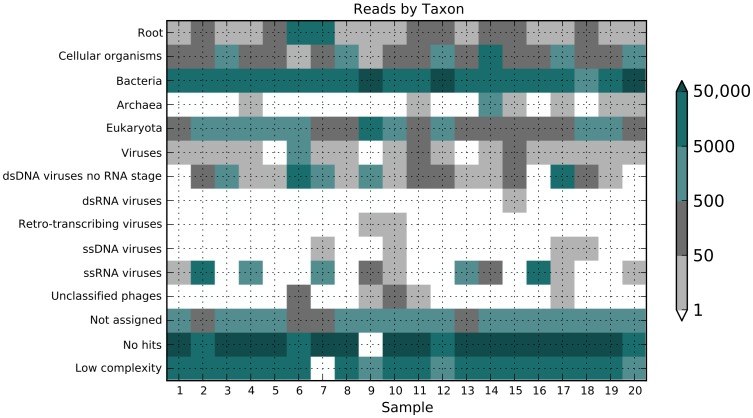
Taxonomy of reads in each sample. 100,000 random reads from each sample dataset were subject to a nucleotide BLASTN search and hits with e values less than 0.001 were collected, and processed with MEGAN4 (see Materials and Methods). The Megan output was processed using Python script to generate a heat map of total reads in each sample in each category. Values were grouped into 5 categories and depicted with the following colors: less than 0 reads, white; 1–50 reads, grey; 51–500 reads, dark grey; 501 to 5000 reads, light green; 5001 to 50,000 reads, green, >50,000 reads, dark green (also see color bar scale to right of figure).

The species content of each sample was obtained by performing a nucleotide BLAST analysis of 100,000 random reads from each sample and parsing the NCBI taxonomic data in the BLAST output using the Lowest Common Ancestor algorithm implemented in MEGAN [28]. A read may share identity in multiple taxa, thus the algorithm places the read in the lowest (most general) taxon that encompasses the set of all identified taxa for that read. At least 5 read hits must occur before a taxon is considered present in the read set. The results of such a taxonomic analysis are presented in Figure 4, organized into 15 taxonomic categories, with colors indicating the number of reads found in each sample for that category (see color bar scale, Figure 4). Several patterns emerge from such an analysis. The viral categories showed the widest range of results. Despite the pretreatment which reduces bacterial material (via centrifugation and DNase treatment) a substantial amount of bacterial sequences were found in these fecal samples (approximately 10%). A large proportion of the reads fail to return a significant BLAST hit with a median of 69,000 of the 100,000 analyzed short reads found in the No Hit category. This may be due to the short sequences, the stringent cutoff used (BLAST Expect value <0.001) and/or the large amount of yet-to-be-characterized micro-organisms in the gut. One important feature becomes clear with such a taxonomic analysis: there is seldom a single species of non-viral material present in these samples. For example, one sample contained *ca.* 80% reads mapping to *Phyllostachys edulis* (a bamboo), and another sample had *ca.* 20% reads mapping to *Sphingobacteria bacterium*, *etc*. Because the samples contents were so varied, a strategy to rapidly remove high frequency, sample-specific, non-viral reads was developed.

### Use SLIM to identify viral sequences

One approach to discovering viruses in deep sequence data is to first remove the abundant reads that dominate the data. These abundant, non-interesting reads are often host or commensal organism ribosomal or repetitive sequences. Manual subtraction of sequences mapping to individual species is possible, but becomes time-consuming when there are multiple species to identify and remove. An iterative search-classify-remove process, SLIM, was developed that addresses this difficulty (outlined in Figure 2). A read in the dataset is subjected to a nucleotide BLAST search, if a significant BLAST hit is returned (Expect value less than 0.001) the full BLAST hit sequence was retrieved from GenBank and a rapid mapping algorithm (MUMmer, [31]) was used to map all reads in the dataset to the GenBank hit (Figure 2). Reads mapping to the GenBank hit were removed from the dataset and categorized as virus or non-virus using identifiers from the GenBank entry. Reads not returning BLAST hits with an Expect value less than the threshold (0.001 used here) were placed into the unknown, mystery category. A new cycle of the process was then initiated with the remaining reads. This process limited the amount of computational time spent identifying and removing sequences from abundantly represented species (e.g. from bacterial ribosomal repeats).

**Figure 5:**
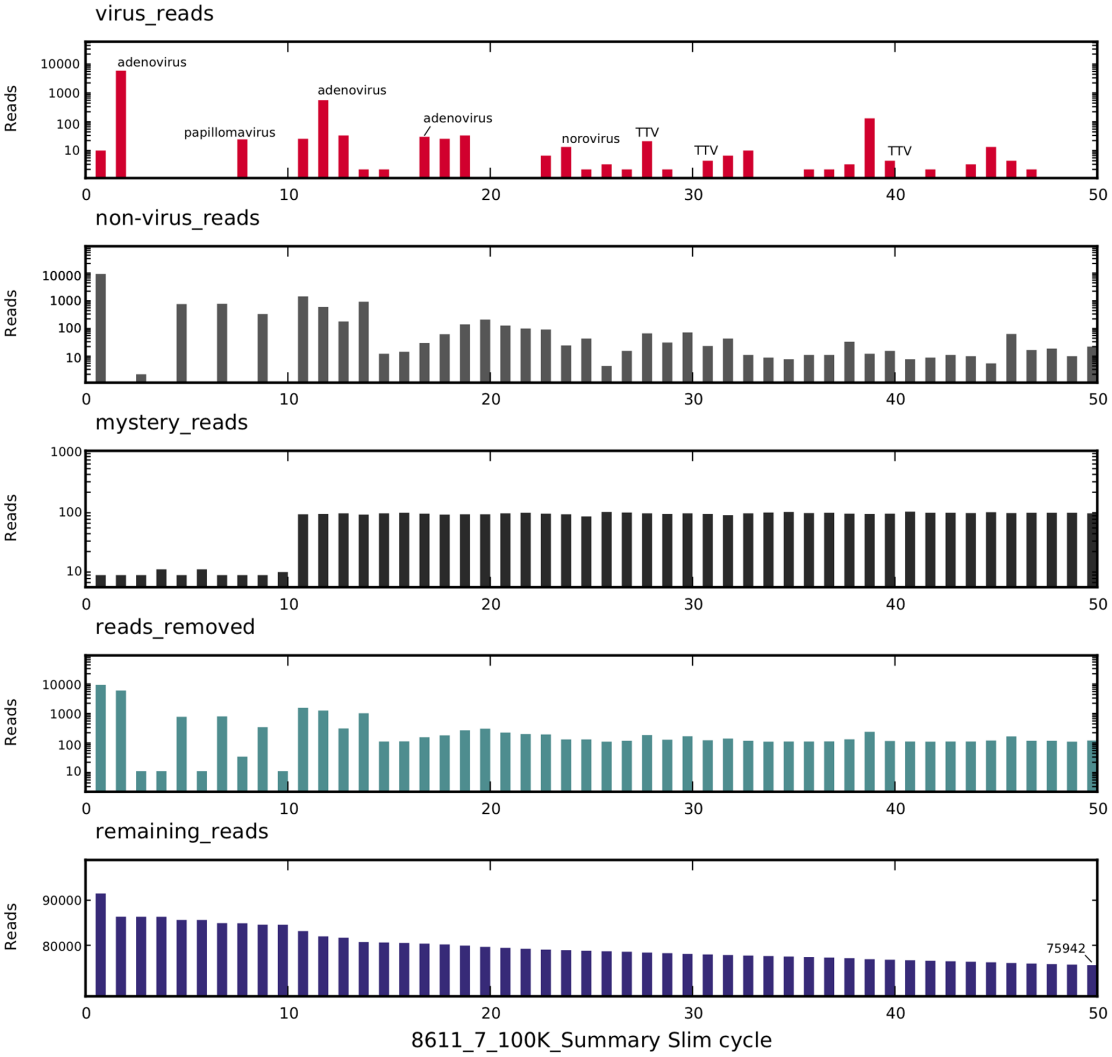
A demonstration of SLIM function. 100,000 random reads reads from sample 17 were processed with SLIM. For each cycle, the number of reads classified as virus, non-virus and mystery, the number of reads removed and the number of reads remaining are plotted. The cycle number and elapsed time is indicated below the graph, the cycle of identification of specific viruses is marked in the upper (virus_reads) graph.

A demonstration of the SLIM process is shown in Figure 5. A random set of 100,000 reads from sample 17 was processed with SLIM. The initial 10 cycles used a batch size of 10 reads, increased to 100 reads/batch from cycles 11–1000 and then 300 reads per cycle thereafter. For each cycle, the numbers of reads classified as virus, non-virus and mystery, the number of reads removed and the remaining reads were plotted. The time required for 50 cycles (removal of ca 24,000 reads) was 95.7 minutes with 252 reads/minute removed. The highest rate of read classification by SLIM occurred in the initial cycles. The average classification rate for cycles 1–10 in this sample was 549 reads/minute for the first 10 cycles. Once the abundant reads were classified and removed, the classification rate per minute fell to that of simple BLASTn searching and SLIM provided no additional advantage (the rate for SLIM cycles 41–50 was 50.3 reads/minute). For comparison, straight BLASTn classification of reads using the same batch sizes and BLASTn settings proceeded at 30 reads/minute and would require 800 minutes to classify all 100,000 reads. Analysis of the entire 100,000 reads are required because the viral reads are distributed randomly throughout the 100,000 read set. If the goal is to identify all virus reads present at sufficient levels for secure virus identification by full genome assembly, straight BLASTn search required 800 minutes while SLIM performed this in 95.7 minutes providing an 8.4 fold increase in processing speed.

The mammalian viruses discovered in the 50 cycles are indicated, while bacteriophage hits that were also collected into the virus bin are shown as non-annotated red bars. Comparing these mammalian virus results to the total mapping data (not shown), it is clear that it is not necessary to perform a BLAST search on each of the 100,000 reads. If a virus is present at sufficient levels for full genome assembly, it should be detected in the first 50 cycles of the SLIM process.

The three adenovirus peaks are a consequence of the stringency used for the mapping step. Each peak is the consequence of the actual read returning a different adenovirus genome after BLASTn searching. For the mapping and removal step in the process, the stringency is set to high level to avoid removing (and possibly misclassifying) weakly homologous reads. We considered it more effective to leave the weaker homology reads in the remaining read set and allow BLAST to identify a closer homology hit at a later step. Thus reads in cycle 2 returned hits to Adenovirus 51 (GenBank JN226765) and Adenovirus 20 (GenBank JN226749) and mapping identified and removed 5315 reads from the set. The cycle 12 peak was due to reads mapping to Human adenovirus 43 (GenBank KC529648) and the mapping identified and removed 295 reads. The cycle 17 peak was due to a read mapping to Human adenovirus 30 (GenBank KF268335). All of the adenovirus BLASTn hits from this sample fall within the same Adenovirus species D as does the *de novo* assembled genomic contig from this sample (Adenovirus_Amsterdam_1995, see below).

**Figure 6:**
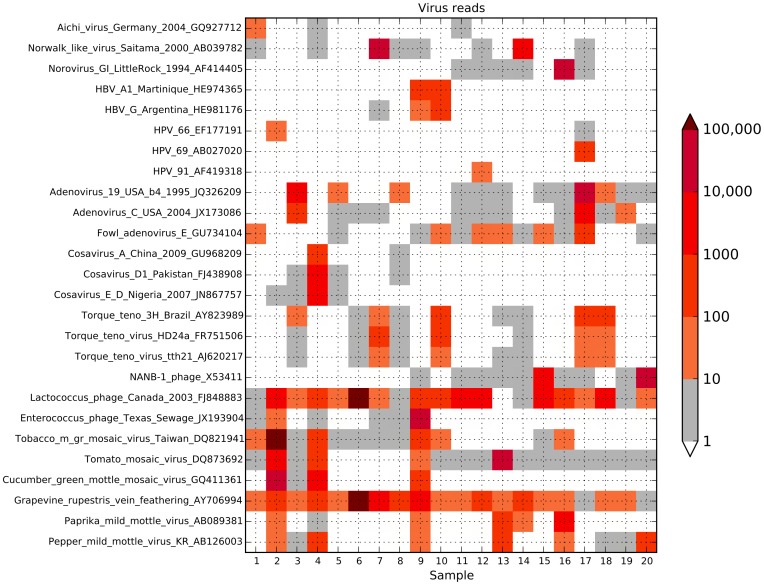
Quantitation of specific virus reads in each of the 20 samples. All reads for each sample (see Table 1 for total number of reads per samples) were mapped to the indicated viral genomes using MUMmer [31]. The number of reads mapped to each virus (normalized for total reads in each sample) is depicted by color (see color bar scale to right of figure).

This iterative classification process was applied to 100,000 random reads from each of the 20 samples to identify the viral sequence content (Figure 6). The total number of reads in each sample mapping to the mammalian viruses aichivirus, adenovirus, cosavirus, hepatitis B virus, human papillomavirus, norovirus and Torque teno virus reads, as well as to select bacteriophage and plant viruses was determined (Figure 6). This identified all bacterial, plant and mammalian viruses in the samples that are available in GenBank. These results were consistent with the more general taxonomic classification provided by MEGAN4 (Figure 4).

### Full genomes of mammalian viruses

**Figure 7:**
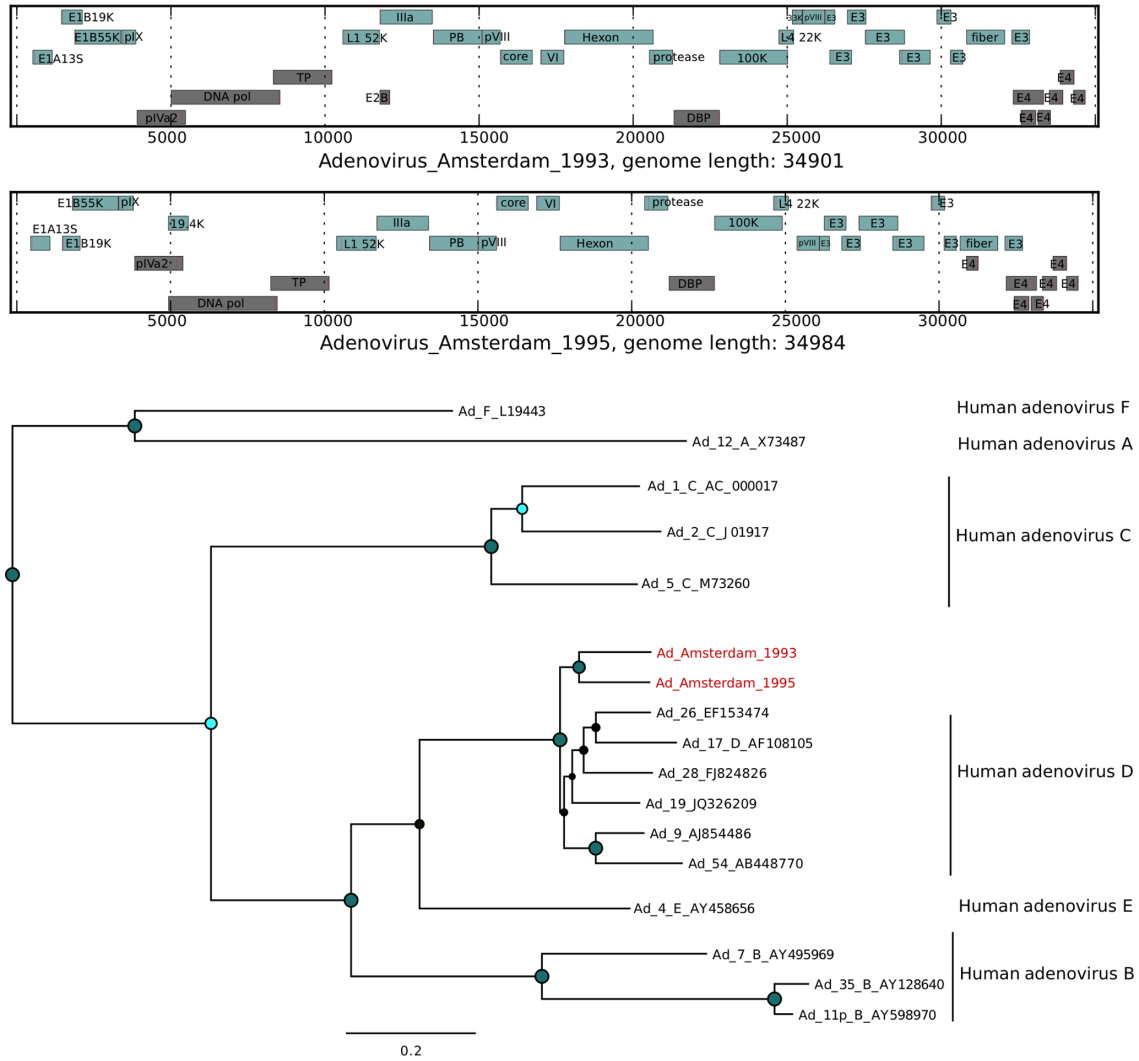
Open reading frame structure and phylogenetic analysis of the adenovirus genomes identified in this study. The ORF pattern of the full genomes grey (for all ORFs >100 amino acids in length), with the initial ATG in each ORF (vertical red bar) and all stop codons (vertical black bars) are indicated. For clarity the stop codon positions were not marked in the adenovirus genomes. Also shown are the maximum likelihood trees inferred using PhyML version 3.0 under the general-time reversible substitution model. Among-site heterogeneity was considered through a discrete-gamma distribution model, and the robustness of the phylogeny assessed through bootstrap analysis of 1000 pseudo-replicates. The trees are marked with green node circles indicating the bootstrap support, (small green circle at 70% support, larger green circle at 100% support, black nodes indicate support below 70%). The genomes identified in this study are marked in red.

**Figure 8:**
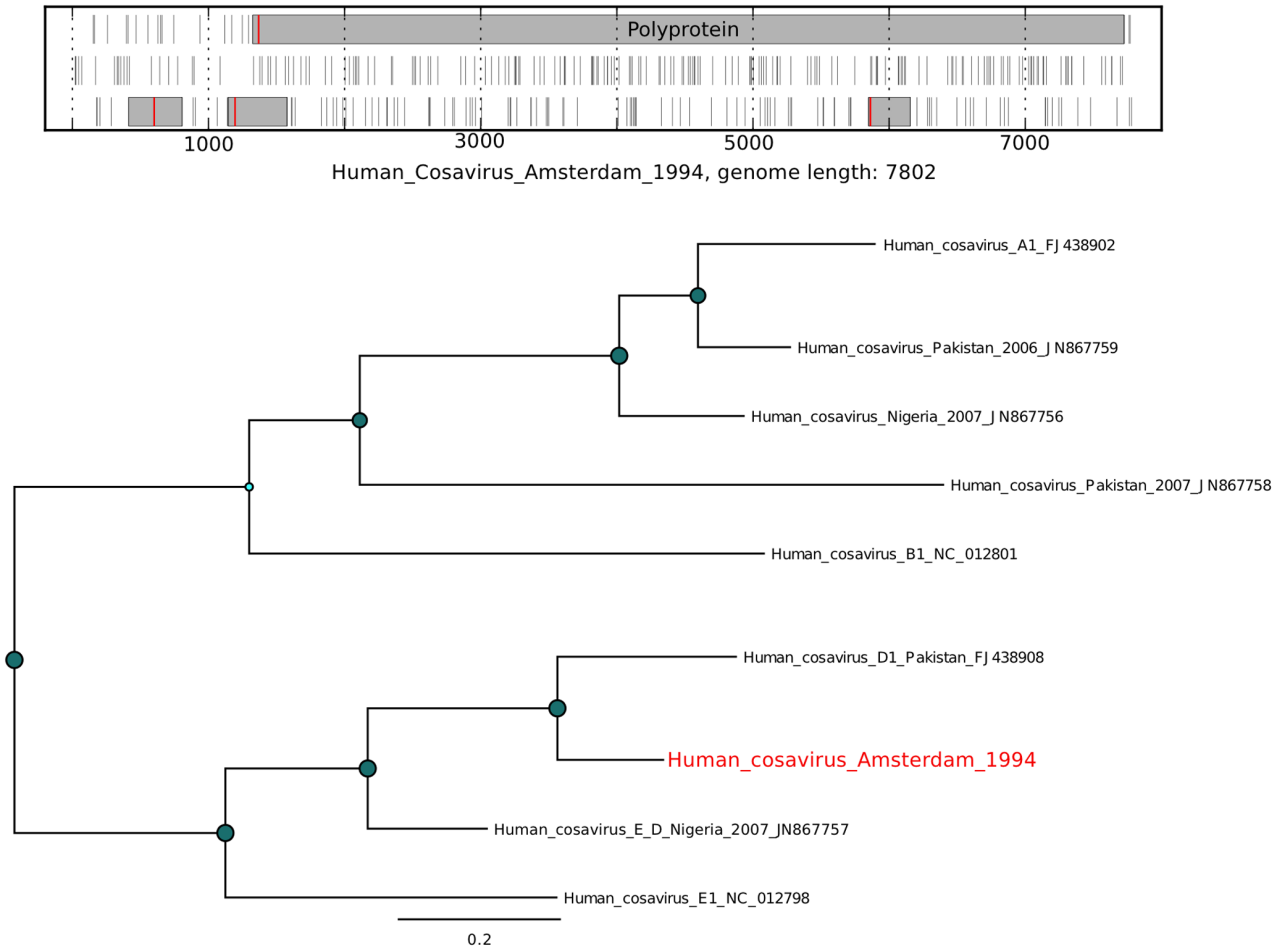
Open reading frame structure and phylogenetic analysis of the human cosavirus genome identified in this study. Analysis and graphical presentation was performed as described in the legend to Figure 7.

**Figure 9:**
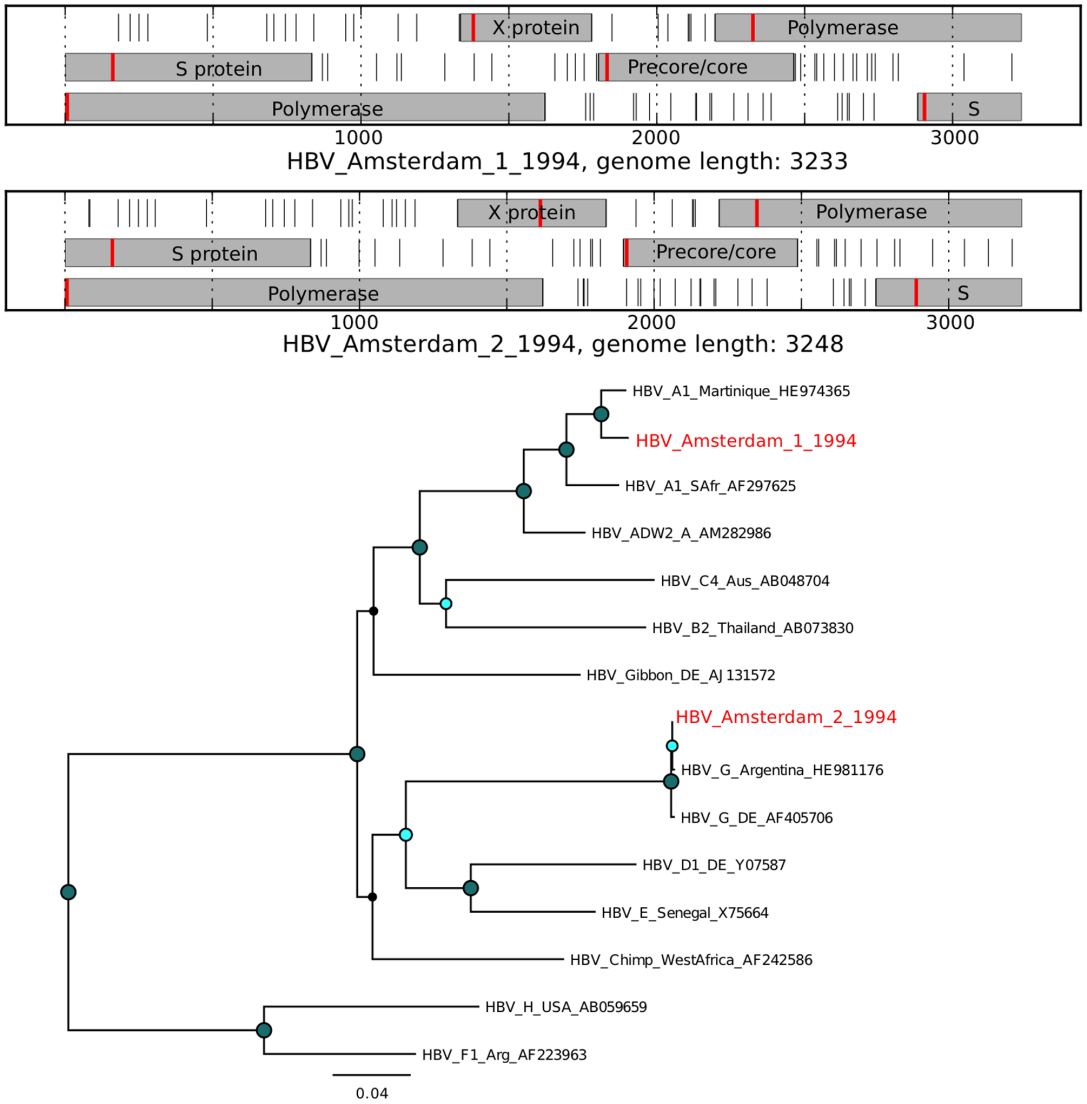
Open reading frame structure and phylogenetic analysis of the hepatitis B virus genomes identified in this study. Analysis and graphical presentation was performed as described in the legend to Figure 7. The HBV reference genome set was from reference [44].

**Figure 10:**
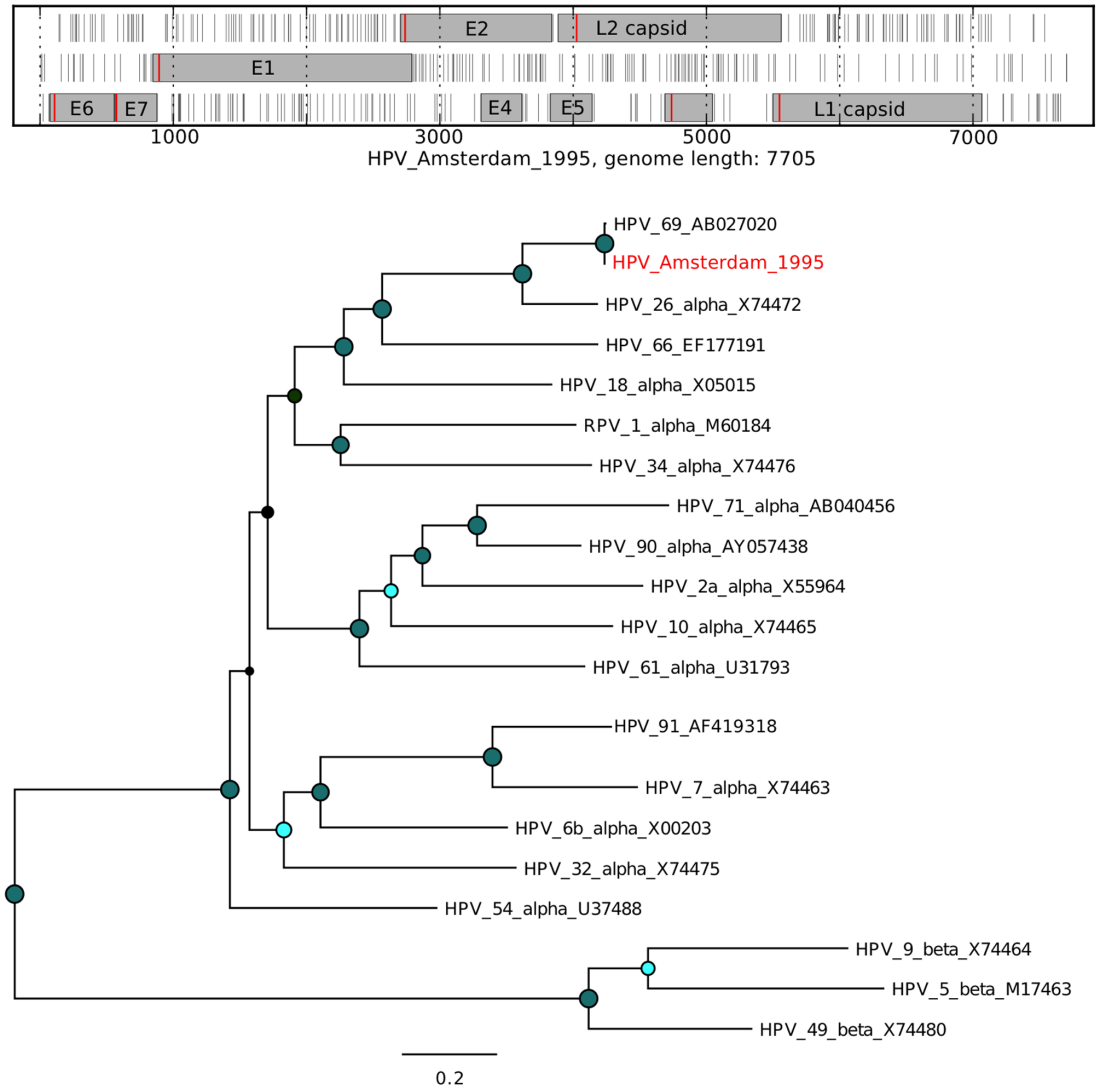
Open reading frame structure and phylogenetic analysis of the human papillomavirus genome identified in this study. Analysis and graphical presentation was performed as described in the legend to Figure 7. The HPV reference genomes are from reference [45], For the phylogenetic analysis, the ORFS for E6-E7-E1-E2-L2-L1 were concatenated.

**Figure 11:**
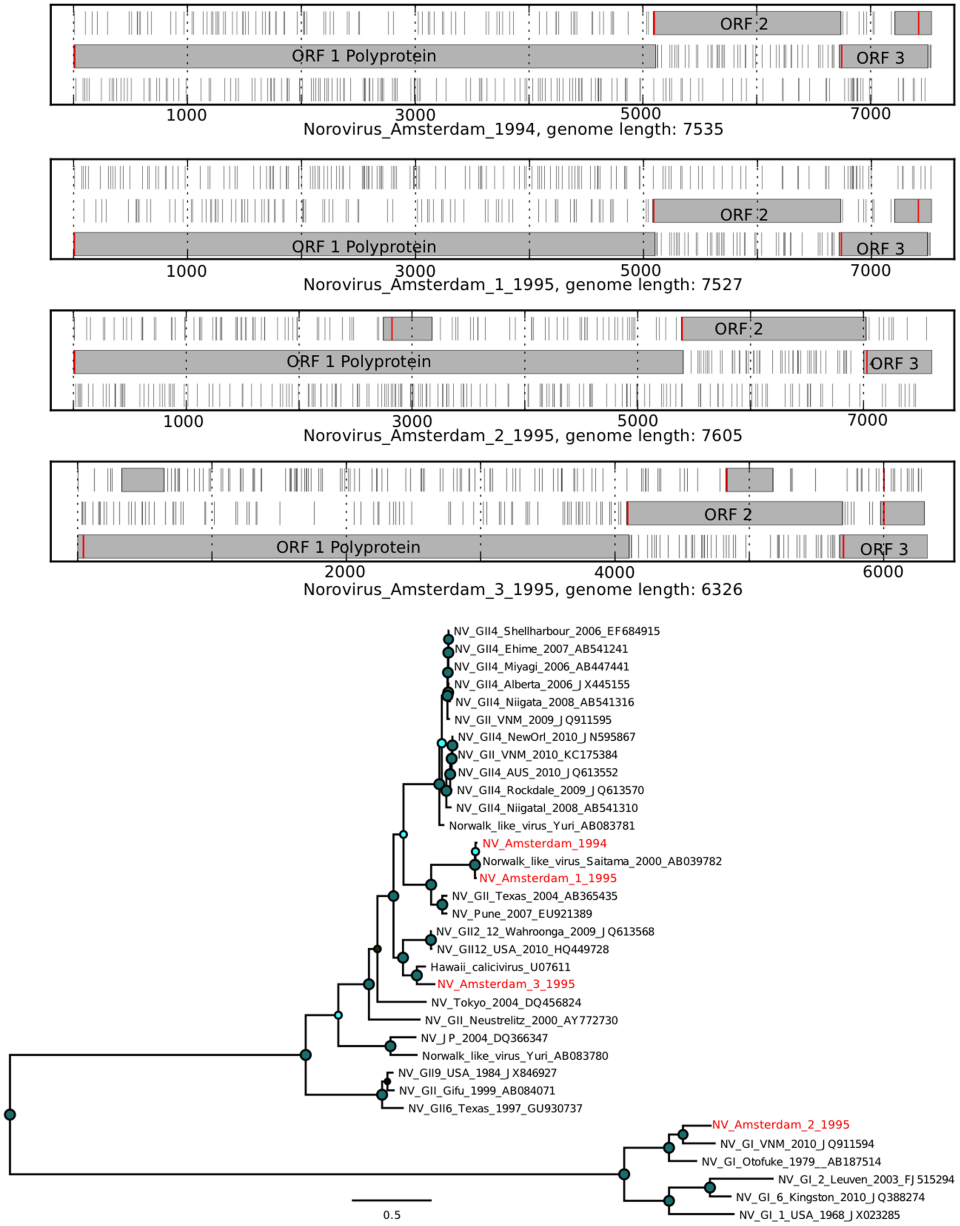
Open reading frame structure and phylogenetic analysis of the norovirus genomes identified in this study. Analysis and graphical presentation was performed as described in the legend to Figure 7.

**Figure 12:**
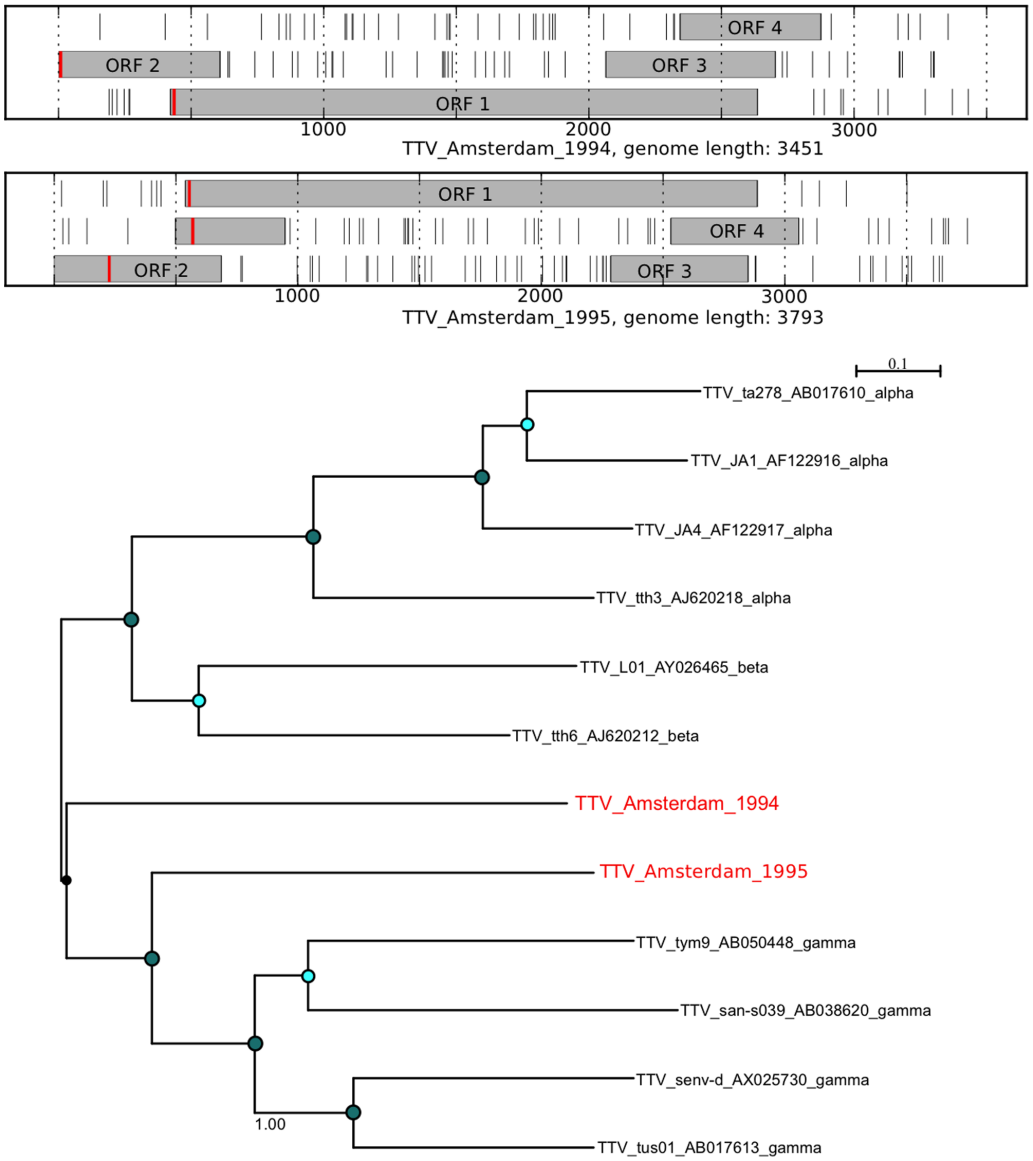
Open reading frame structure and phylogenetic analysis of the Torque teno virus genomes identified in this study. Analysis and graphical presentation was performed as described in the legend to Figure 7. The TTV reference set was from reference [34].

For several mammalian viruses, a substantial number of short reads were identified. Assembly of complete genomes from these data was performed using *de novo* assembly with SPAdes [30]. An analysis of the novel virus open reading frames (ORFs) in comparison with the expected ORFs for a given virus was performed to assess the validity of the assembly (Figures 7, 8, 9, 10, 11, and 12). The novel genome or sub-genomic region was aligned to a broad set of reference sequences, and maximum-likelihood (ML) phylogenetic trees were inferred (Figures 7, 8, 9, 10, 11, and 12).

In total, 12 full genomes of mammalian viruses could be generated. Viruses belonged to the RNA and DNA viruses (cosavirus, adenovirus, hepatitis B virus, human papillomavirus, norovirus, and torque teno virus).

Human adenovirus reads could be detected in 16 of the 20 samples (Figure 6) with sufficient coverage in samples 3 and 17 for assembly into complete genomes (Adenovirus_Amsterdam_1993, Adenovirus_Amsterdam_1995). The adenovirus genome, although large, is double-stranded DNA and this may account for the high recovery rate in the sequence set. Analysis of the open reading frame structures of the assembled genomes shows the expected adenovirus ORFs (Figure 7). The two adenovirus genomes cluster within the group D adenoviruses, consistent with an enteric infection (Figure 7).

Cosavirus is a new genus in the *Picornaviridae* family first described in 2008 [32] [33]. Consistent with the frequent identification of this virus in fecal samples, cosavirus sequences were detected in 5 of the 20 samples (Figure 6). Reference-based mapping of the sample 4 reads to either of the two closest cosavirus genomes was unable to generate a complete genome (results not shown), however *de novo* assembly of the sample 4 reads generated a full length genome (Cosavirus_Amsterdam_1994) with the expected conserved features of a cosavirus (Figure 8). The genome has a low G/C content of 43.15%, similar to that reported for cosavirus (43.8%, [32]). A methionine codon at nucleotide position 746 is within a standard Kozak sequence (RNNAUGG : AATATGG) and a short stretch poly-pyrimidine is found just upstream (TTTTCCTTTT). Cosaviruses typically encode a single large open reading frame that is translated into a polyprotein and processed into 11 proteins. The Cosavirus_Amsterdam_1994 genome shows the expected large open reading frame of a cosavirus (Figure 8) with the predicted protease cleavage sites conserved relative to other cosaviruses (results not shown). According to the classification proposed by Kapusinszky et al. [33], Cosavirus_Amsterdam_1994 can be considered a new genotype within the D species, since the VP1 amino acid sequence is less than 88% identical to the other 5 cosavirus D-serotypes, while phylogenetically it clearly clusters within the cosavirus group D (Figure 8). As expected, the recombinant E2/D cosavirus (Nigeria_2007_JN867757) is phylogenetically placed between species D and E, when performing the analysis on the whole genome. According to our analysis, the closest relative to Cosavirus_Amsterdam_1994 is genotype D1 (VP1 AA sequence identity: 69.1%) and the most distant is genotype D3 (54.9%), while the identity range between all the other genotypes is 52.4–63.9%. To our knowledge, only 5 genotypes within the cosavirus D species are known (D1 to D5, excluding the E2/D recombinant whose VP1 belongs to species E). The new genotype described here is the first member of genotype D6. Interestingly, between the only 2 complete genomes of species D (D1 and D6), the recombinant cosavirus E2/D is more closely related to serotype D6 than to serotype D1 (AA identity after the breakpoint of E2/D vs. D1 is 94.3%, while vs. D6 is 96.8%).

Hepatitis B viral sequences were detected in 3 samples (Figure 6). The HBV reads in samples 9 and 10 could be assembled into complete viral genomes with the expected HBV ORF structure (HBV_Amsterdam_1_1994, HBV_Amsterdam_2_1994). HBV_Amsterdam_1_1994 clusters with HBV genotype A1, HBV_Amsterdam_2_1994 clusters with HBV genotype G (Figure 9). The presence of HBV in fecal samples has been previously observed [10].

Human papillomavirus sequences were detected in 5 samples (Figure 6), with a partial genome from sample 12 (*ca.* 60% genome coverage, results not shown) and a complete genome assembled from sample 17 (HPV_Amsterdam_1995, Figure 10). HPV_Amsterdam_1995 clustered with HPV type 7 and type 91 alpha-papillomaviruses and shows the expected ORFs for this virus (Figure 10).

Norovirus sequences were detected in 14 of the 20 samples (Table 1, Figure 6) with genomes assembled from samples 7, (NV_Amsterdam_1994) sample 14 (NV_Amsterdam_1_1995), sample 16 (NV_Amsterdam_2_1995) and sample 17 (NV_Amsterdam_3_1995). NV_Amsterdam_1994 and NV_Amsterdam_1_1995 clustered with a Norwalk-like virus from 2000. NV_Amsterdam_2_1995 clusters with the GI noroviruses, while NV_Amsterdam_3_1995 is most closely related to an older calicivirus from Hawaii (Figure 11). All four noroviruses showed the expected three large open reading frames (Figure 11).

Infection with Torque teno virus (TTV) was detected in 14 of the 20 samples (Figure 6) consistent with the ubiquitous human distribution of this virus. *De novo* assembly allowed the generation of 5 TTV contigs 2500 nt or larger. The larger heterogeneity in TTV [34] make it difficult to determine if the shorter contigs are authentic genomes or fragments, so we report only the two full genome-sized contigs from sample 6 (TTV_Amsterdam_1994) and sample 17 (TTV_Amsterdam_1995). Analysis of the open reading frame structures of the assembled genomes shows the expected TTV ORFs (Figure 12), the two genomes are more closely related to the diverse gamma TTV [34], [35].

### Bacteriophages

A large number of reads mapping to plant viruses and to bacteriophages were identified. The bacteriophage and plant viral content of the mammalian gut virome has been described previously [36], [12]. Lactococcus phage frequently infects the bacterial cultures used in cheese production [37] and can be a frequent dietary component as a result of cheese consumption. Lactococcus phage sequences were identified in 19 of the 20 samples. Two samples contained sufficient material for complete genomes (sample 6, 178041 reads, sample 11, 12634 reads) although a proper analysis of these genomes is beyond the scope of this study.

A virus associated with non-A, non-B hepatitis (NANB-1) was identified in patients in the early 1980s [38], [39], [40], [41]. The disease potential of NANB-1 is still controversial and the virus is likely to be a bacteriophage [13]. NANB-1 was detected in two of the fecal samples (sample 15, sample 20) and sufficient sequence was available to assemble a partial (80%, sample 15) or a complete (sample 20) genomes.

Sequences mapping to an Enterococcus phage were identified in several samples, sufficient reads were present in sample 9 to assembly a genome-sized contig.

### Plant viruses

All samples contained at least one plant virus and most contained sequences from several plant viruses. Complete genome-sized contigs related to Tobacco mild green mosaic virus (TMGMV), Paprika mild mottle virus, Cucumber green mottle mosaic virus, Tomato mosaic virus (TMV) and a partial genomes of Grapevine rupestris vein (GRV) feathering virus were assembled from the data. These plant viruses are not known to be associated with human disease and their presence may simply reflect recent dietary consumption.

## Discussion

The novel process reported here, ViSeq, provides a quantitative picture of the viral content of human fecal samples. The method shows detection sensitivity approaching real-time PCR assay levels, however without *a priori* sequence information on the target viruses. For many of the viruses detected, the depth of sequencing provided by the method allows assembly of full viral genomes. In order to process the increased quantity of sequence data generated by this method, we have developed a virus identification algorithm, SLIM which employs iterative cycles of BLAST identification, clustering and read removal. The combined ViSeq and SLIM method facilitated the identification and assembly of 12 complete mammalian virus genomes (including a previously unknown cosavirus genotype) from the 20 fecal samples without prior sequence data on these viruses.

The goal of the virus detection is to provide sufficient evidence that a virus is present in a sample and we believe that full genome assembly is a useful standard. One can calculate that a 5000 nt virus genome at 10 fold coverage requires 50,000 nt of sequence or 333, 149 nt reads. 333 reads in a dataset of 1 million reads corresponds to 33 reads in 100,000 (0.033%) and would be a good practical cutoff for reliable virus detection.

The SLIM algorithm provides a useful tool for virus detection work. The algorithm does not require large amounts of computer memory and can be run locally on a laptop as long as internet access is available for the BLAST and GenBank calls. One obvious step for future improvement lies in the handling of the BLAST unidentified reads. The current virus identification methods, including those described in this work, rely heavily on the data in GenBank. However, a large fraction of the sequence data generated here was not classified by nucleotide BLAST searches. Malboeuf *et al*. [42] also noted a similar high level of unknown sequences (48–95%) in their analysis of viral samples, highlighting the challenges of identifying short read data using BLAST. Future efforts will focus on alternate virus identification to characterize these novel sequences.

Recently it has been hypothesized that the enteropathy associated with AIDS may be a consequence of elevated replication of enteric viruses promoted by the immune system decline late in AIDS [12]. This in turn may lead to leakage of bacterial endotoxin and T-cell activation promoting further HIV-1 replication. The diversity of the fecal virome during SIV infection showed a much higher level of vertebrate viral genome content and diversity in pathogenic SIV infection (Rhesus monkey) compared to non-pathogenic SIV infection (African Green Monkey) [12]. A number of potentially enteropathic viruses showed elevated levels during pathogenic SIV infection, including adenovirus. Consistent with the non-human primate data, adenovirus, HPV, HBV and norovirus were identified in sufficient levels in HIV-1 infected human fecal samples for full genome assembly. The methods described here would provide a sensitive method of tracking the fecal virome during AIDS progression and could reveal the replication of pathogenic viruses as a consequence of immune decline.
