# Correction: Full Genome Virus Detection in Fecal Samples Using Sensitive Nucleic Acid Preparation, Deep Sequencing, and a Novel Iterative Sequence Classification Algorithm

**DOI:** 10.1371/journal.pone.0101921

**Published:** 2014-07-01

**Authors:** The PLOS ONE Staff

The second author’s name is incorrect. The correct name is Bas B. Oude Munnink. The correct citation is: Cotten M, Oude Munnink BB, Canuti M, Deijs M, Watson SJ, et al. (2014) Full Genome Virus Detection in Fecal Samples Using Sensitive Nucleic Acid Preparation, Deep Sequencing, and a Novel Iterative Sequence Classification Algorithm. PLoS ONE 9(4): e93269. doi:10.1371/journal.pone.0093269

## References

[1] Cotten M, Oude Munnink B, Canuti M, Deijs M, Watson SJ, et al (2014) Full Genome Virus Detection in Fecal Samples Using Sensitive Nucleic Acid Preparation, Deep Sequencing, and a Novel Iterative Sequence Classification Algorithm. PLoS ONE 9(4): e93269 doi:10.1371/journal.pone.0093269 24695106 10.1371/journal.pone.0093269 PMC3973683

